# Prolonged Alprazolam Treatment Alters Components of Glutamatergic Neurotransmission in the Hippocampus of Male Wistar Rats—The Neuroadaptive Changes following Long-Term Benzodiazepine (Mis)Use

**DOI:** 10.3390/ph16030331

**Published:** 2023-02-21

**Authors:** Marina Zaric Kontic, Milorad Dragic, Jelena Martinovic, Katarina Mihajlovic, Zeljka Brkic, Natasa Mitrovic, Ivana Grkovic

**Affiliations:** 1 Department of Molecular Biology and Endocrinology, VINČA Institute of Nuclear Sciences—National Institute of the Republic of Serbia, University of Belgrade, 11351 Belgrade, Serbia; 2 Laboratory for Neurobiology, Department of General Physiology and Biophysics, Faculty of Biology, University of Belgrade, 11158 Belgrade, Serbia; 3 Department of Biomedicine, Aarhus University, 8000 Aarhus, Denmark

**Keywords:** Alprazolam, benzodiazepines, hippocampus, *N*-Methyl-d-aspartate receptor, gammaaminobutyric acid receptor type A, excitatory amino acid transporters 1/2

## Abstract

Alprazolam (ALP), a benzodiazepine (BDZ) used to treat anxiety, panic, and sleep disorders, is one of the most prescribed psychotropic drugs worldwide. The side effects associated with long-term (mis)use of ALP have become a major challenge in pharmacotherapy, emphasizing the unmet need to further investigate their underlying molecular mechanisms. Prolonged BDZ exposure may induce adaptive changes in the function of several receptors, including the primary target, gammaaminobutyric acid receptor type A (GABA_A_R), but also other neurotransmitter receptors such as glutamatergic. The present study investigated the potential effects of prolonged ALP treatment on components of glutamatergic neurotransmission, with special emphasis on N-Methyl-D-aspartate receptor (NMDAR) in the hippocampus of adult male Wistar rats. The study revealed behavioral changes consistent with potential onset of tolerance and involvement of the glutamatergic system in its development. Specifically, an increase in NMDAR subunits (NR1, NR2A, NR2B), a decrease in vesicular glutamate transporter 1 (vGlut1), and differential modulation of excitatory amino acid transporters 1 and 2 (EAAT1/2, in vivo and in vitro) were observed, alongside a decrease in α1-containing GABA_A_R following the treatment. By describing the development of compensatory actions in the glutamatergic system, the present study provides valuable information on neuroadaptive mechanisms following prolonged ALP intake.

## 1. Introduction

Alprazolam (ALP) is a highly potent benzodiazepine (BDZ) and one of the most prescribed psychotropic medications worldwide, used for the treatment of anxiety, panic, and sleep disorders [1,2]. Long-term use of ALP and other BZDs is not recommended but is commonly practiced due to the complexity of the aforementioned disorders, which often require prolonged treatment that exceeds the manufacturer guidelines [3]. Alprazolam also exerts a high risk of abuse due to its rewarding, disinhibitory and anxiolytic effects, which is why it is often taken for prolonged periods and/or in high doses [1,4,5]. Regardless of the reason, prolonged ALP treatment is almost always harmful and causes side effects such as tolerance, dependence, and withdrawal syndrome [3].

Alprazolam produces sedative, anxiolytic, muscle relaxant, anticonvulsant, and cognition impairing effects by binding to the BDZ site located at the junction of the α/γ subunits of gammaaminobutyric acid (GABA) receptor type A (GABA_A_R). As a positive allosteric modulator, ALP increases the affinity of GABA_A_R for GABA [6,7] indirectly inducing adaptive changes in the function of various receptors and consequently different side effects. The most prominent side effect is the development of tolerance to BDZ actions, which has been associated with changes in GABA_A_R subunit expression, assembly, or allosteric subunit uncoupling [8]. Experimental data suggest that BDZ tolerance, as well as dependence and withdrawal following abrupt discontinuation of prolonged treatment, may also be associated with an enhanced glutamatergic transmission [8]. The ability of N-Methyl-D-aspartate receptor (NMDAR) antagonists to block the development of tolerance and signs of dependence after long-term treatment with diazepam was demonstrated almost three decades ago, indicating the importance of NMDAR-dependent mechanisms in the development of BDZ tolerance [9,10]. To date, however, there is no experimental evidence to support a generalized conclusion regarding the role of glutamatergic neurotransmission in the development of BDZ tolerance and other side effects [7,11,12].

Alprazolam and all other BDZs modulate neurotransmission in various brain structures, as GABA_A_Rs are distributed throughout the whole CNS, with high densities in cortex, hippocampus (HIP), cerebellum, amygdala, and basal ganglia in both human and rat brain [7,13,14,15]. Among these structures, HIP represents a unique interface between cognition and emotion [16]. This brain structure is involved not only in cognitive processes such as episodic memory and spatial navigation, but also in the pathogenesis of mood and anxiety disorders [17], as it is functionally heterogeneous along its dorsoventral axis [17,18].

In summary, although ALP and other BZDs are very effective in relieving the indicated symptoms, its recommended use is limited to several weeks due to the high potential for tolerance development and other side effects such as dependence and withdrawal symptoms [3]. Nevertheless, prolonged use for months or even decades has been observed in many users. Prolonged BZD use has been associated with deterioration of cognitive function, increased risk of dementia and dementia-like illness, impaired sensory and motor function, aggressive behavior, and others [3]. Short-acting BZDs such as ALP appear to be at greater risk for adverse effects and tend to exhibit greater dependence [3]. Although ALP is one of the most commonly (mis)used BZDs, the exact nature of the resulting synaptic changes is limited and has been unfairly neglected. Considering the above risks of prolonged use of BDZs, the present study investigated the previously unknown effects of 14 days ALP treatment on glutamatergic components, mainly NMDAR, in HIP of adult male Wistar rats.

## 2. Results

### 2.1. Effect of Prolonged ALP Treatment on Anxiety—Like Behavior

#### 2.1.1. Open Field Test

**Figure 1:**
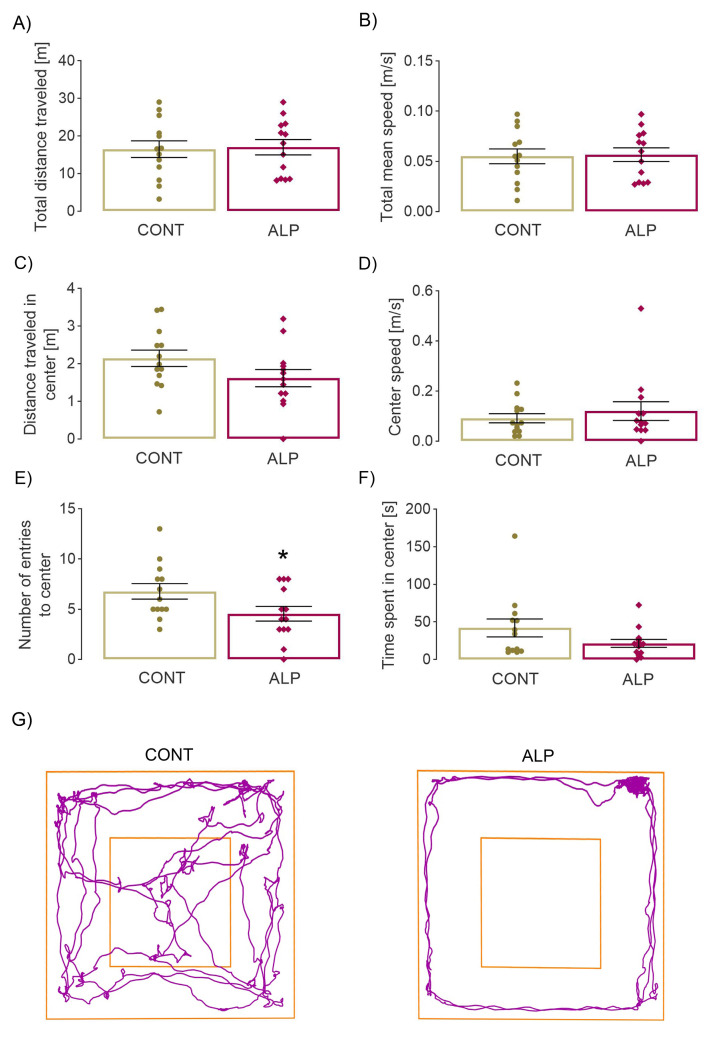
Effect of prolonged 14 days ALP treatment on behavioral parameters in open field test. (**A**) Total distance traveled in arena [m], (**B**) total mean speed (m/s), (**C**) distance traveled in center [m], (**D**) mean speed in center [m/s], (**E**) number of entries to center, (**F**) time spent in center [s], (**G**) representative track plots of VEH (left) and ALP (right) group. Data were statistically analyzed by two-tailed *t*-test and expressed as percentage of the mean values of CONT group ± SEM (dots in the graphs represent values of individual animals). Symbols indicate significant differences between CONT and ALP group: * *p* < 0.05; animals per group: 13.

The two-tailed *t*-test revealed a statistically significant decrease in the number of entries into the center of the arena (*p* = 0.046, *t* = 2.103, Figure 1E) in the ALP group compared to VEH, while the time spent in it remained the same (Figure 1F), as did the distance traveled in the center and speed (Figure 1C–D, respectively). No changes were observed in other parameters reflecting overall locomotor performance, such as total distance traveled and speed (Figure 1A–B). The results obtained, especially the lower number of entries in the ALP group compared with VEH, as well as the track plots (Figure 1G), could indicate tolerance development.

#### 2.1.2. Elevated Plus Maze Test

**Figure 2:**
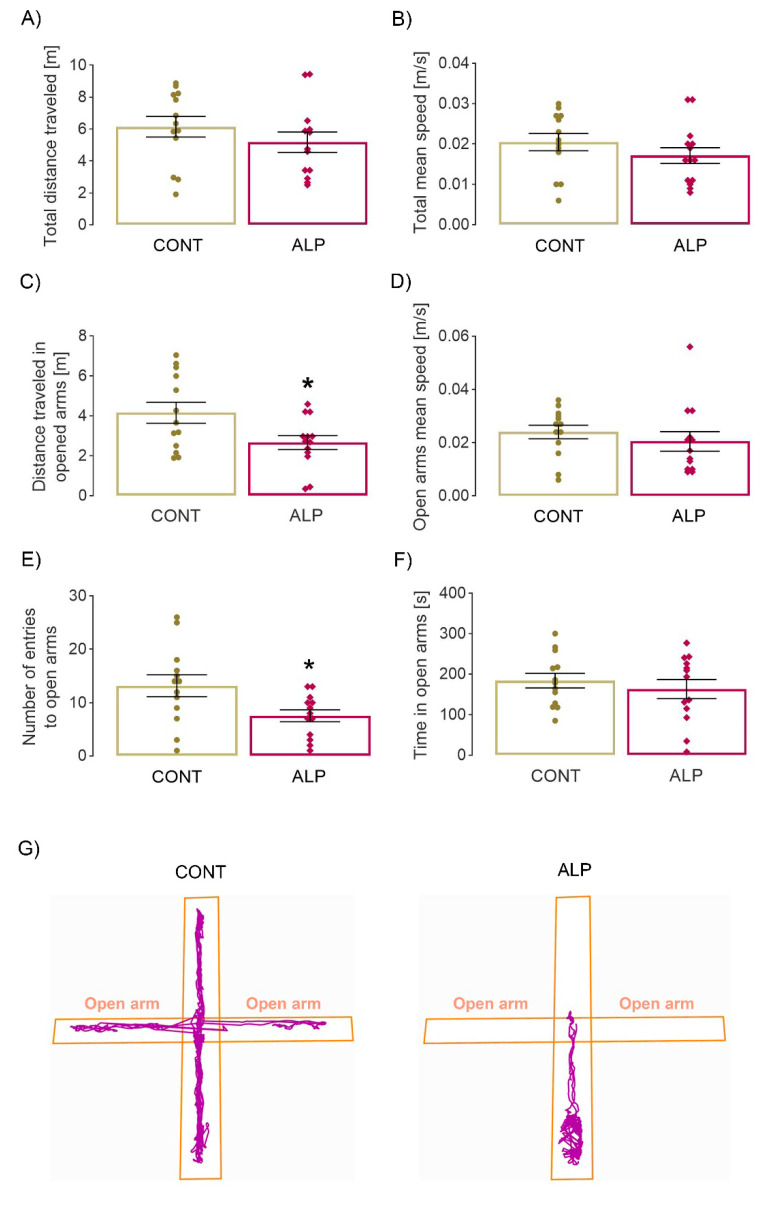
Effect of prolonged 14 days ALP treatment on behavioral parameters in elevated plus maze test. (**A**) Total distance traveled in maze [m], (**B**) total mean speed [m/s], (**C**) distance traveled in open arms [m], (**D**) mean speed in open arms [m/s], (**E**) number of entries to open arms, (**F**) time spent in open arms [s], (**G**) representative track plots of VEH (left) and ALP (right) group. Data were statistically analyzed by two-tailed *t*-test and expressed as percentage of the mean values of CONT group ± SEM (dots in the graphs represent values of individual animals). Symbols indicate significant differences between CONT and ALP group: * *p* < 0.05; animals per group: 13.

The two-tailed *t*-test revealed a statistically significant decrease in the number of entries into the open arm (*p* = 0.024, *t* = 2.41, Figure 2E, in agreement with the representative track plots in Figure 2G), with no change in time spent (Figure 2F). However, the distance traveled in open arms was also lower in animals treated with ALP compared with control animals (*p* = 0.0273, *t* = 2.350). Other parameters studied, such as total distance and total/open arms speed, remained unchanged (Figure 2A,B,D). Similar to the results of open field test, the obtained data suggest a possible anxiogenic effect of prolonged ALP treatment, which could be caused by the development of tolerance.

### 2.2. Effect of Prolonged ALP Treatment on Protein Expression of GARA_A_R α1 Subunit

**Figure 3:**
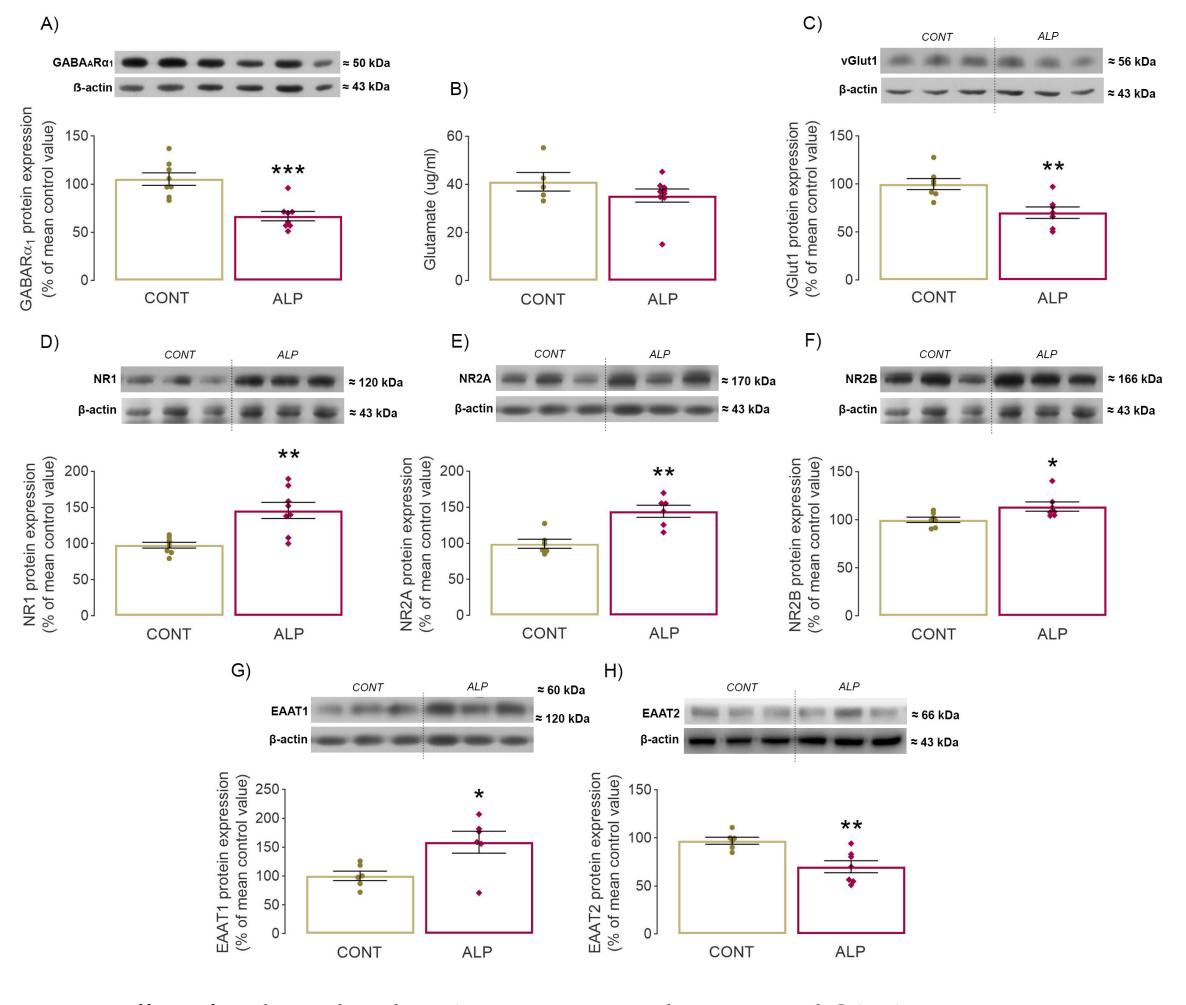
Effect of prolonged 14 days ALP treatment on hippocampal GABA_A_Rα1 protein expression and components of glutamatergic signaling in vivo. (**A**) Representative immunoblot and quantitative data of Western blot analysis of GABA_A_Rα1 protein expression, (**B**) glutamate level in hippocampal P2 fraction (µg/mL), (**C**–**H**) representative immunoblots and quantitative data of Western blot analysis of target proteins expression in hippocampal P2 fraction: (**C**) vGlut1, (**D**) NR1, (**E**) NR2A, (**F**) NR2B, (**G**) EAAT1, (**H**) EAAT2. Data were statistically analyzed by two-tailed *t*-test and expressed as percentage of the mean values of CONT group ± SEM (dots in the graphs represent values of individual animals). Symbols indicate significant differences between CONT and ALP group: * *p* < 0.05, ** *p* < 0.01, *** p < 0.001; animals per group: 8.

Given that the ALP binding site is located on the junction of α/γ subunits, protein expression of GARA_A_Rα1 was investigated. Statistical analysis revealed a decreased level of GARA_A_Rα1 in the ALP group compared to CONT (*p* = 0.0003, *t* = 4.728, Figure 3A, Supplementary Figure S1).

### 2.3. Effect of Prolonged ALP Treatment on Components of Glutamatergic Signaling

The first step taken to assess potential changes in the glutamatergic system following ALP treatment involved examining the neurotransmitter glutamate. ALP had no effect on hippocampal glutamate levels compared to CONT (*p* = 0.244, *t* = 1.224, Figure 3B, Supplementary Figure S1).

ALP treatment decreased protein expression of vesicular glutamate transporter 1 (vGluT1) in the ALP group compared to CONT (*p* = 0.004, *t* = 3.584, Figure 3C). A significant effect of ALP was also detected on protein expression of all three investigated NMDAR subunits. The two-tailed *t*-test showed a significant increase in NR1 (*p* = 0.001, *t* = 4.071, Figure 3D, Supplementary Figure S1), NR2A (*p* = 0.002, *t* = 4.284, Figure 3E, Supplementary Figure S1) and NR2B subunit protein expression (*p* = 0.029, *t* = 2.486, Figure 3F, Supplementary Figure S1) in the ALP group compared with CONT. Analysis of two major glutamate transporters, EAAT1 and EAAT2, also revealed significant alterations in the hippocampal P2 fraction. Protein expression of EAAT1 (*p* = 0.018, *t* = 2.812, Figure 3G, Supplementary Figure S1) was increased, whereas levels of EAAT2 were decreased in animals treated with ALP (*p* = 0.004, *t* = 3.575, Figure 3H) compared to CONT.

### 2.4. Effect of 48-h ALP Treatment on Astrocytes’ Viability

**Figure 4:**
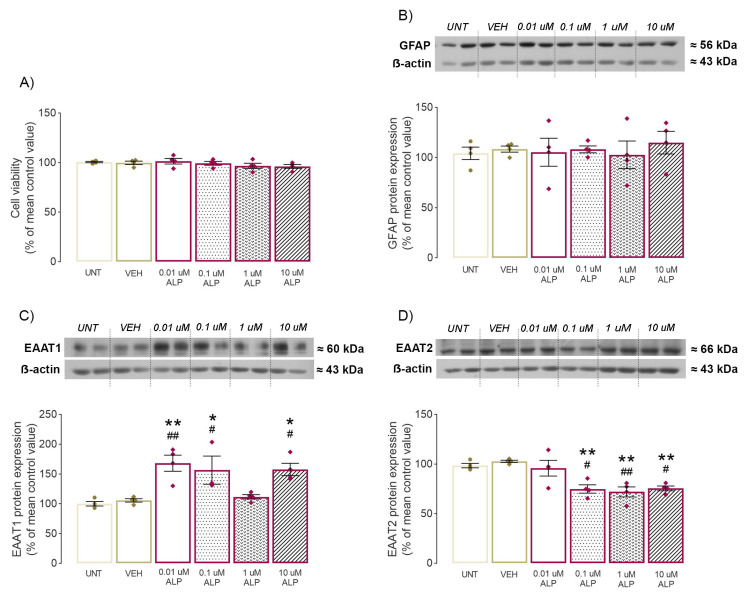
Effect of 48-h treatment with increasing ALP doses on primary cortical astrocyte culture. (**A**) Results of MTT assay used for determination of cell viability, (**B**–**D**) representative immunoblots and quantitative data of Western blot analysis of target proteins expression whole cell fraction of primary cortical astrocyte culture: (**B**) GFAP, (**C**) EAAT1, (**D**) EAAT2. Data were statistically analyzed by two-tailed *t*-test (**A**) or one-way ANOVA (**B**–**D**) and expressed as percentage of the mean values of CONT group ± SEM (dots in the graphs represent values of individual animals). Symbols indicate significant differences between respective group and CONT (* *p* < 0.05, ** *p* < 0.01) or VEH group (# *p* < 0.05, ## *p* < 0.01); samples per group: 4.

The first step in the in vitro part of the research was to evaluate the potential effects of VEH or increasing doses of ALP on astrocyte viability using the MTT assay. Neither of the applied treatments provoked alterations of astrocytes’ viability in the MTT assay (ANOVA summary: *F* = 0.9934, *p* = 0.4492, Figure 4A).

### 2.5. Effect of 48-h ALP Treatment on GFAP Protein Expression In Vitro

Since reactive astrocytes are characterized by high-level expression of glial fibrillary acidic protein (GFAP) [19], its protein expression was examined in primary astrocyte culture. Protein levels remained unchanged in all experimental groups, indicating that neither vehicle nor ALP had any effect, regardless of dose administrated (ANOVA summary: *F* = 0.197, *p* = 0.959, Figure 4B, Supplementary Figure S1).

### 2.6. Effect of 48-h ALP Treatment on EAAT1/2 Protein Expression In Vitro

Given that excitatory amino acid transporters 1 and 2 (EAAT1/2) are mainly expressed on astrocytes [20] and that their alteration was detected in the hippocampus of ALP treated rats, further analyses were performed on primary astrocyte cultures using increased ALP concentrations. First, no significant difference in EAAT1/2 protein expression was observed between the UNT and VEH groups (*p* = 0.9987, *p* = 0.9809, Figure 4C–D, respectively, Supplementary Figure S1). However, ALP treatment had significant effects on both EAAT1 (*F* = 8.541, *p* = 0.0003, Figure 4C, Supplementary Figure S1) and EAAT2 protein expression (*F* = 9.867, *p* = 0.0001, Figure 4D, Supplementary Figure S1). Consistent with in vivo results, the post hoc test revealed a significant increase in EAAT1 following 0.01 µM (*p* = 0.0025, *p* = 0.0056), 0.1 µM (*p* = 0.0233, 0.0471) and 10 µM ALP treatment (*p* = 0.0111, *p* = 0.0242) compared to both UNT and VEH, respectively (Figure 4C, Supplementary Figure S1). Surprisingly, no difference was observed in the 1 µM ALP group (*p* = 0.9639, *p* = 0.9982) compared to UNT and VEH, respectively (Figure 4C, Supplementary Figure S1). Conversely, EAAT2 protein expression was lowered after 0.1 µM (*p* = 0.0148, *p* = 0.0034), 1 µM (*p* = 0.00550, *p* = 0.0013) and 10 µM ALP treatment (*p* = 0.0181, *p* = 0.0042) compared to UNT and VEH, respectively (Figure 4D, Supplementary Figure S1). However, the lowest ALP dose, 0.01 µM, did not provoke any significant differences compared to UNT and VEH, respectively (*p* = 0.9978, *p* = 0.8674, Figure 4D, Supplementary Figure S1).

## 3. Discussion

### 3.1. “Crosstalk between Behavior and GABA_A_R”—Changes in Anxiety-Like Behavior Together with Significant GARA_A_Rα1 Decrease following Prolonged ALP Treatment

The behavioral assessment indicated potential increase in anxiety-like behavior in the ALP group compared with CONT as evidenced in decreased center and open arms entries. The observed decrease in distance traveled in open arms may also be indicative as the increased anxiety-like behavior manifested as the enhanced “fear” of moving in elevated and open spaces. In support of this, the absence of changes in total distance traveled and speed confirms that the observed changes cannot be simply attributed to a decrease in overall locomotor activity. However, other parameters such as the time spent in center/open arms remained unaltered. This could point to potential anxiogenic effect of prolonged ALP treatment, but also emphasize that obtained data should be interpreted with caution.

The observed behavioral changes may be the result of tolerance onset caused by prolonged ALP administration. It should be stressed, however, that literature data on anxiety-like behavior and the occurrence of tolerance remain inconsistent, partly due to differences in dosing, treatment route, and chosen behavioral paradigm and overall experimental design. Nevertheless, the development of tolerance following prolonged BDZ treatment has been clearly showed in many studies using various behavioral tests in rodents [21,22,23,24]. Similar results were shown by Duke and coworkers (2021) in primates, who demonstrated that chronic ALP treatment leads to physical dependence as well as tolerance to some behavioral effects in female rhesus monkeys. Their results also supported the hypothesis that GABA_A_Rα1 is involved in the development of tolerance to some behavioral aspects and withdrawal signs following chronic ALP treatment and BDZ in general [25]. Indeed, one of the common mechanisms established in terms of regulation of the net neurons’ responsiveness to repeated drugs exposure, including BDZ, is the decrease in the target receptor numbers, in this case—GABA_A_R [7,11]. Most consistent changes following prolonged BDZ in both humans and animal models include alterations in the expression of α1, 2, 4, and γ2 subunits of GABA_A_R [26]. According to the literature, prolonged BDZ exposure may cause changes in GABA_A_R subunits composition, increased internalization, changes in the receptor’s phosphorylation state and the uncoupling of the GABA/BDZ binding site [11,24,26]. It is possible that the observed decrease in GABA_A_Rα1 is the result of reduced subunit expression and/or increased internalization following prolonged ALP treatment. Regardless of the mechanism, this alteration suggests the occurrence of adaptive changes in the function of the receptor that could strongly influence overall neuronal plasticity.

### 3.2. Changes in Glutamatergic Neurotransmission following Prolonged ALP Treatment—Alterations of NMDAR Subunits, vGlut1 and EAAT1/2 Levels

Neuronal excitability is the result of a delicate balance between opposing neurotransmitter systems—the inhibitory GABAergic and the excitatory glutamatergic system. Functionally, the inhibitory effects of GABA may be balanced by the excitatory effects of glutamate as the two neurotransmitter systems are metabolically linked through their synthetic intermediate glutamine [27]. Given these tight connections between the GABAergic and glutamatergic systems, the aim of the study was to assess the changes in the components of glutamatergic neurotransmission following prolonged ALP exposure.

**Figure 5:**
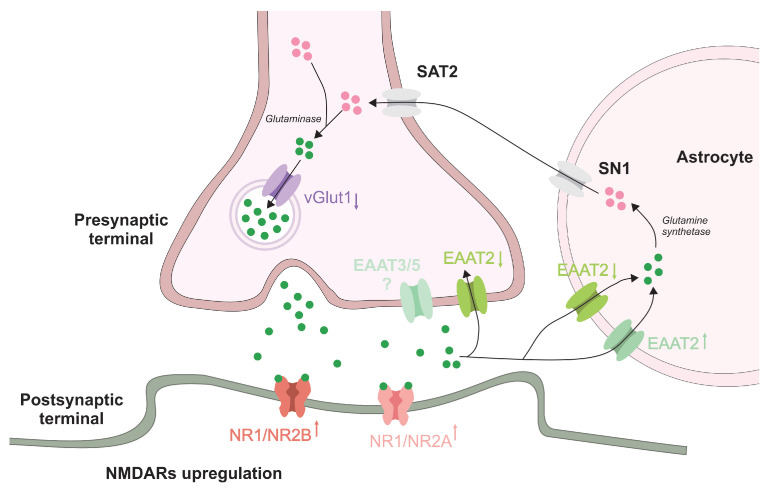
Proposed mechanism of effect of prolonged ALP treatment on components of glutamatergic signaling. ALP induces the increase of NMDAR subunits on post-synaptic membrane and influence the glutamate balance in the synaptic cleft trough differential modulation of EAAT1/2 in astrocytes and/or neurons and decrease of vGlut1 in pre-synaptic terminals. SN1—System N1, SAT2—System A Transporter 2, EAAT3/5—excitatory amino acid transporters 3/5.

The prolonged ALP treatment resulted in profound changes in glutamatergic neurotransmission. The decrease in vGlut1, which mediates the uploading of glutamate into synaptic vesicles, was associated with increase in NMDAR subunit (NR1, NR2A, and NR2B) in the ALP group compared with CONT. The up growth of constitutively expressed NR1 subunit, together with NR2A and NR2B, may indicate an overall increase in the number of membrane NMDARs. In view of this, it is possible to assume that previously reported NMDAR—induced neuroadaptive changes following prolonged BDZ usage [7,28,29,30] may be governed by enhanced number of inserted receptors rather than changes in receptor subunit composition and downstream signaling (Figure 5). As mentioned earlier, glutamatergic sensitization may play an important role in the development of BDZ tolerance, consistent with the observed changes in glutamatergic components. It has been demonstrated that many of the behavioral properties of BDZ also apply to antagonists of excitatory amino acid receptors [11]. NMDAR antagonists have been shown to be effective in animal models of anxiety exerting anticonvulsant, sedative, amnestic, and muscle relaxant effects [31,32]. Importantly, the similarities in pharmacology between BDZ and NMDAR antagonists do not extend to non-NMDA receptor antagonists, although a greater proportion of rapid excitatory transmission in the adult CNS is attributable to the actions of glutamate at non-NMDAR such as α-amino-3-hydroxy-5-methyl-4-isoxazolepropionic acid receptors (AMPAR) and kainate receptors [33].

The results regarding EAAT1/2 protein expression following ALP treatment, both in vivo and in vitro, suggest that the two transporters are modulated differently. To date, data on the effects of prolonged BDZ usage have only been reported in the study by Palamada and coworkers (2002), who also demonstrated differential modulation of EAAT1 and EAAT2 in vitro with respect to their uptake capacity [34]. EAAT1/2 are mainly expressed on astrocytes, although EAAT2 is also found on neurons. These transporters represent critical components of glutamatergic transmission because they control the level of extracellular glutamate and, indirectly, the glutamatergic firing rate [12,20]. They also prevent uncontrolled and sustained activation of glutamate receptors, allow the recycling of the transmitter, and thus provide protection from hyperexcitability and excitotoxicity. The observed alterations in EAAT1/2 protein expression may affect all components of glutamatergic signaling and contribute to the occurrence of side effects characteristic for long-term ALP usage. Since EAAT2 is the major glutamate transporter in the brain, responsible for more than 90% of glutamate uptake [12], its decrease could indicate decreased glutamate uptake and increased glutamate levels in the synaptic cleft. However, the potential rise in synaptic glutamate could be counteracted by observed increase in EAAT1 and decrease in vGlut1 which would provide a compensatory mechanism that could maintain the level of glutamate in the synaptic cleft stable, as observed in the present study.

## 4. Materials and Methods

All experimental procedures were approved by the Ethics Committee for the Use of Laboratory Animals of Vinca Institute of Nuclear Sciences—National Institute of the Republic of Serbia, University of Belgrade, Belgrade, Republic of Serbia (license number: 323-07-06136/2020-05) following the compliance with the European Communities Council Directive (2010/63/EU) for animal experiments, according to the guidelines of the EU registered Serbian Laboratory Animal Science Association, a member of the Federation of the European Laboratory Animal Science Associations. Care was taken to minimize pain and discomfort of the animals and all experimental procedures were carried out in accordance with the UK Animals (Scientific Procedures) Act 1986 and associated guidelines and, National Institutes of Health guide for the care and use of Laboratory animals (NIH Publications No. 80-23, revised 1996).

### 4.1. Experimental Groups and Treatment—In Vivo

The experiments were carried out using adult 2.5 months old male Wistar rats obtained from a local colony. Animals (3 per cage, total number = 56) were housed under standard conditions: 12 h light/dark regime, ad libitum access to commercial rat pellet and tap water, constant ambient temperature (21 ± 2 °C) and humidity. The rats were randomly divided into 2 experimental groups: (1) CONT group—control animals injected with 2% Tween 80 (Sigma-Aldrich, St. Louis, MI, USA) in normal saline (used as a solvent for ALP), and (2) ALP group—rats treated with ALP (Galenika, Serbia, 2 mg/kg). Both VEH and ALP were administered i.p. during 14 days between 08:00 and 09:00. The dose of ALP was selected according to the preliminary results of the pilot study (results not showed). The pilot study also included the determination of possible effects of vehicle administration, i.e., potential differences between intact and vehicle-treated animals. No differences were found between the two with respect to the proteins and glutamate levels studied (results not shown), so only the two groups mentioned were included in the formal analysis. Fourteen days lasting ALP treatment was selected given the two following facts: a) long-term use of BDZs is defined as two or more months of usage [3], b) 14 rat days correspond approximately to 1.3 human years (10.5 rat days = 1 human year, for adult rat, [35].

### 4.2. Behavioral Testing

All animals (total number = 26, 13 per group) were placed in the testing room 30 min prior to the testing to allow acclimatization. The test arenas were cleaned with 70% alcohol solution after each testing to eliminate odor cues. 

### 4.3. Open Field Test

The open field apparatus consisted of a square arena (100 cm × 100 cm × 40 cm) divided into 16 equal squares (dimensions of each square were 25 cm × 25 cm). During the test, each rat was placed in the center of the arena and recorded during 5 min. The videos were then quantified using ANY-maze Video Tracking System 7.11 software. The total distance traveled, and mean speed, were used to determine total locomotor activity and exploration. Anxiety-like behavior was estimated from the time spent inside four central compartments of the arena (overall dimensions of all four compartments were 50 cm × 50 cm) and the number of entries in these compartments.

### 4.4. Elevated Plus Maze Test

The apparatus consisted of two open arms (40 cm L × 10 cm W) and two enclosed arms (40 cm L × 10 cm W × 40 cm H), branching from a central square platform (10 cm × 10 cm). The test was performed by placing a rat on the central platform of the maze, facing one of the open arms, and letting it move freely. Each session lasted 5 min. The behavior was continuously videotaped by a camera placed above the apparatus during 5 min period and quantified using ANY-maze Video Tracking System 7.11 software. The total distance traveled alongside mean speed were used to determine total locomotor activity, while the number of entries in open arms and the time spent in opened arms were used to determine anxiety-like behavior.

### 4.5. Preparation of Crude Synaptosomal P2 fraction In Vivo

The animals (*n* = 16, 8 per group) were decapitated following behavioral testing (Harvard Apparatus, USA). Brains were removed and HIPs were dissected for preparation of crude synaptosomal fractions (P2). Tissue samples were placed in 10 vol of ice cold 0.32 M sucrose in 5 mM Tris-HCl buffer (pH 7.4) and homogenized manually in a Teflon/glass homogenizer (clearance 0.20 mm) by 20 gentle up- and down-strokes. Crude nuclear fraction and cell debris were removed by centrifugation at 1000× *g* for 10 min. The resulting supernatant was transferred and centrifuged at 10,000× *g* for 20 min to obtain the crude synaptosomal pellet which was re-suspended in 5 mM Tris-HCl, pH 7.4. Isolated crude synaptosomal P2 fraction contains bulk of the synaptosomes, free mitochondria, but also other membrane fragments deriving from glial cells and neurons [36]. The protein concentration was estimated using modified method of Lowry [37].

### 4.6. Western Blot Analysis—In Vivo

The Western blot analysis was performed using previously prepared hippocampal P2 fraction (8 per group). Equal amount of total protein from each sample diluted in Laemmli sample buffer (250 mM Tris–HCl pH 6.8, 10% SDS, 30% glycerol, 5% β-mercaptoethanol, 0.02% bromophenol blue) was separated on 8% or 10% SDS-PAGE gel, depending on molecular weight of the target protein, and transferred to PVDF membranes (Imobilion-P membrane, Millipore, Burlington, MA, USA). Membranes were blocked in TBS containing 5% non-fat milk (Sigma-Aldrich, USA) and 0.1% Tween 20 (Sigma-Aldrich, USA) for 1 h and incubated overnight at 4 °C with primary antibodies (Table 1). The next day, membranes were incubated with appropriate horseradish peroxidase conjugated secondary antibody (Table 1). After washing in TBST, the membranes were incubated with the enhanced chemiluminescence system (Immobilon Western Chemiluminescent HRP Substrate, Millipore, USA) and immunoreactive bands were detected on X-ray films in the dark chamber, scanned and saved in .tiff format. Β-Actin was used as a loading control. The signal intensity was evaluated using Image J software. The analysis was performed at least 3 times for each target protein to ensure the reliability of the results.

### 4.7. Examination of Glutamate Level

Levels of hippocampal free glutamate (5 *per* CONT group; 9 *per* ALP group) were measured using a colorimetric glutamate assay kit (Abcam, UK, ab83389) according to the manufacturer’s specifications and expressed as µg of glutamate *per* mL of sample. The animals included in this analysis were not subjected to behavioral testing.

### 4.8. Experimental Groups and Treatment—In Vitro

Given that in vivo results indicated a significant alteration of the two proteins mainly expressed on astrocytes—EAAT1/2 [20], the next part of the research was directed toward evaluation of these protein in isolated astrocytes in vitro. Six experimental groups were included in in vitro investigation: UNT—untreated astrocytes, VEH (sterile dH2O containing 0.02% Tween 80 added to the medium), and four groups treated with increasing concentrations of ALP named as 0.01 µM ALP, 0.1 µM ALP, 1 µM ALP and 10 µM ALP. Primary astrocyte culture was prepared from cerebral cortex of 2 days old male Wistar rats (*n* = 4 *per* group), as previously described [38]. Cerebral cortices were dissected in cold phosphate buffer solution (PBS) and mechanically dissociated in Dulbecco’s modified Eagle medium (DMEM+, Sigma-Aldrich, USA) under sterile conditions. Cell suspension was centrifuged three times at 500× *g* for 4 min and after second centrifuge/washing step, cells were passed through 21G (ø 0.8 mm) and 23G (ø 0.6 mm) sterile needles. Finally, cells were resuspended in DMEM supplemented with 10% fetal bovine serum (FBS, Gibco, USA), 1 mmol/L sodium pyruvate (Sigma-Aldrich, USA), 100 IU/mL penicillin (Gibco, USA) and 100 μg/mL streptomycin [Gibco, USA, (DMEM+)]. Cells were then seeded on 60 mm sterile Petri dishes and grown at 37 °C in a humified incubator with 5% CO2. DMEM+ was replaced every two days favoring the growth of astrocytes, while residual cell types were washed off under pressure. After reaching 80% confluence, cells were washed in PBS twice, detached using 0.025% trypsin, 0.02% EDTA in PBS, seeded and grown on new 60 mm sterile Petri dishes. Purity of astrocytes in obtained culture is considered to be more than 98% [37]. When reaching confluence, cells were trypsinized again, counted and seeded on 24-well plate for MTT assay and on 6-well plate for Western blot analysis with density of 2 × 104 cells/cm2. The astrocyte cultures were maintained to reach 90% confluence and then treated with different concentrations of ALP (0.01, 0.1, 1 and 10 µM) for 48 h. Alprazolam was first dissolved in sterile dH2O containing 0.02% Tween 80 and then added to the cells in DMEM+ growth medium. After the 48 h (timeframe chosen based on authors’ previous experience with in vitro experiments), treatment medium was removed, and the astrocytes were washed in PBS twice. For the isolation of total proteins, cells from each well were scraped and resuspended in 140 µL of lysis buffer containing 150 mM NaCl, 50 mM Tris-HCl, 1 mM EDTA and 1% NP-40 detergent. The suspension was centrifuged at 10,000× *g* on 4 °C for 15 min to obtain the whole cell fraction, supernatant was collected, and protein concentration was determined using BCA Protein Assay Kit (Thermo Scientific, Waltham, MA, USA), according to the manufacturer’s instructions.

### 4.9. Western Blot Analysis—In Vitro

An equal amount of total protein from each sample (4 per group) diluted in Laemmli sample (375 mM Tris-HCl, pH 6.8, 12% sodium dodecyl sulfate, 60% glycerol, 0.03% bromophenol blue, 5% β-mercaptoethanol) was separated on 10% SDS-PAGE gel and protein expression of EAAT1/2 and GFAP was analyzed as described in the text above (Subsection Western blot in vivo and Table 1). 

### 4.10. Microculture Tetrazolium Assay

Microculture tetrazolium assay (MTT assay) was used for assessment of cellular viability in vitro based on the measurement of total mitochondrial activity. After the 48 h treatment, medium in each well was replaced by 200 µL of MTT solution in a concentration of 0.5 mg/mL and cells were left to incubate on 37 °C for 30 min. Formazan crystals, formed as a result of metabolic activity of viable cells, were then dissolved by adding of 720 µL of DMSO. Samples from each well (200 µL) were placed in triplicate on the 96-well plate and absorbance was measured on 570 nm using WALLAC 1420-Victor2 Multilabel Counter (PerkinElmer, Waltham, MA, USA).

### 4.11. Statistical Analysis

The two-tailed *t*-test or one-way analysis of variance (one-way ANOVA) followed by Tukey’s post-hoc test were performed for statistical comparison among groups. The results were presented as mean ± SEM values or percent of mean ± SEM of CONT group. The values of *p* < 0.05 were considered statistically significant. For all statistical analyses and graphical presentation, GraphPad Prism 9.0 (San Diego, CA, USA) software package was used.

## 5. Conclusions and Future Prospects

The present study demonstrated that prolonged ALP treatment affects both GABA_A_R and components of glutamatergic neurotransmission, possibly contributing to the observed signs of tolerance development. The alteration of glutamatergic components following ALP treatment including the increase in NMDAR subunits, the decrease in vGlut1 and the differential modulation of EAAT1/2 may collectively represent a compensatory mechanism attributable to the sustained suppression of glutamatergic neurons by enhanced inhibitory impulses from GABAergic neurons. The presented data provide valuable and, to our knowledge, the first information on components of glutamatergic neurotransmission following prolonged ALP treatment. Further research is needed to examine the observed changes in more detail with other aspects of glutamatergic signaling.

To avoid any overstatement, the authors would like to point out the limitations of the study, such as fractions used, which limit the conclusions regarding the proteins studied (e.g., extra-synaptic vs. synaptic) as well as glutamate levels, “narrow” gender, and species/strain aspect (study was performed only on male Wistar rats). Further studies incorporating the aforementioned variables are planned, as well as research focusing on other brain structures, particularly the amygdala.

## Figures and Tables

**Table 1:** List of primary and secondary antibodies used in Western blot analysis.

Antigen	Manufacturer and Catalog No	Species and Dilution
NR1	Cell Signaling Techonology, USA, #5704 RRID: AB_1904067	rabbit monoclonal; 1:1000
NR2A	Merck Millipore, USA, #07-632 RRID: AB_310837	rabbit monoclonal; 1:1000
NR2B	Abcam, UK, 93610 RRID: AB_10561972	mouse monoclonal, 1:4000
vGluT1	Abcam, UK, 134283 RRID: AB_2923539	mouse monoclonal, 1:4000
EAAT1	Cell Signaling Techonology, USA; # 5684T RRID: AB_10671594	rabbit monoclonal; 1:1000
EAAT2	Abcam, UK; ab69098 RRID: AB_2190732	rabbit monoclonal; 1:1000
GABA_A_R α_1_	Sigma Aldrich, USA, G4416 RRID: AB_477016	rabbit monoclonal; 1:5000
β-actin	Thermo Fisher Scientific, USA; PA1-21167 RRID: AB_557422	rabbit polyclonal; 1:5000
mouse IgG	R&D systems, bio-techne, USA; HAF007 RRID: AB_357234	goat polyclonal; 10,000
rabbit IgG	Invitrogen, USA; # 31460 RRID: AB_228341	goat polyclonal; 1:10,000

## Data Availability

Data is contained within the article.

## Supplementary Materials

The following supporting information can be downloaded at: https://www.mdpi.com/article/10.3390/ph16030331/s1, Figure S1: original (unprocessed) images of representative blots.

## Abbreviations

ALP	alprazolam
AMPAR	α-amino-3-hydroxy-5-methyl-4-isoxazolepropionic acid receptor
BDZ	benzodiazepine
EAAT1	excitatory amino acid transporter 1
EAAT2	excitatory amino acid transporter 2
GABA	gammaaminobutyric acid
GABA_A_R	gammaaminobutyric acid receptor type A
GABA_A_Rα1	α1 subunit of gammaaminobutyric acid receptor type A
HIP	hippocampus
MTT assay	microculture tetrazolium assay
NMDAR	N-Methyl-D-aspartate receptor
NR1	type 1 subunit of N-Methyl-D-aspartate receptor
NR2A	type 2A subunit of N-Methyl-D-aspartate receptor
NR2B	type 2B subunit of N-Methyl-D-aspartate receptor
vGluT1	vesicular glutamate transporter 1

## References

[1] Ait-Daoud N., Hamby A.S., Sharma S., Blevins D. (2018). A Review of Alprazolam Use, Misuse, and Withdrawal. J. Addict. Med..

[2] Willems I.A., Gorgels W.J., Oude Voshaar R.C., Mulder J., Lucassen P.L. (2013). Tolerance to benzodiazepines among long-term users in primary care. Fam. Pract..

[3] Edinoff A.N., Nix C.A., Hollier J., Sagrera C.E., Delacroix B.M., Abubakar T., Cornett E.M., Kaye A.M., Kaye A.D. (2021). Benzodiazepines: Uses, Dangers, and Clinical Considerations. Neurol. Int..

[4] Hedegaard H., Bastian B.A., Trinidad J.P., Spencer M., Warner M. (2018). Drugs Most Frequently Involved in Drug Overdose Deaths: United States, 2011–2016. Natl. Vital Stat. Rep..

[5] George T.T., Tripp J. (2022). Alprazolam. StatPearls.

[6] McKernan R.M., Rosahl T.W., Reynolds D.S., Sur C., Wafford K.A., Atack J.R., Farrar S., Myers J., Cook G., Ferris P. (2000). Sedative but not anxiolytic properties of benzodiazepines are mediated by the GABA(A) receptor alpha1 subtype. Nat. Neurosci..

[7] Vinkers C.H., Olivier B. (2012). Mechanisms Underlying Tolerance after Long-Term Benzodiazepine Use: A Future for Subtype-Selective GABA(A) Receptor Modulators?. Adv. Pharmacol. Sci..

[8] Izzo E., Auta J., Impagnatiello F., Pesold C., Guidotti A., Costa E. (2001). Glutamic acid decarboxylase and glutamate receptor changes during tolerance and dependence to benzodiazepines. Proc. Natl. Acad. Sci. USA.

[9] Steppuhn K.G., Turski L. (1993). Diazepam dependence prevented by glutamate antagonists. Proc. Natl. Acad. Sci. USA.

[10] Van Sickle B.J., Cox A.S., Schak K., Greenfield L.J., Tietz E.I. (2002). Chronic benzodiazepine administration alters hippocampal CA1 neuron excitability: NMDA receptor function and expression. Neuropharmacology.

[11] Allison C., Pratt J.A. (2003). Neuroadaptive processes in GABAergic and glutamatergic systems in benzodiazepine dependence. Pharmacol. Ther..

[12] Rodriguez-Campuzano A.G., Ortega A. (2021). Glutamate transporters: Critical components of glutamatergic transmission. Neuropharmacology.

[13] Braestrup C., Albrechtsen R., Squires R.F. (1977). High densities of benzodiazepine receptors in human cortical areas. Nature.

[14] De Blas A.L., Vitorica J., Friedrich P. (1988). Localization of the GABAA receptor in the rat brain with a monoclonal antibody to the 57,000 Mr peptide of the GABAA receptor/benzodiazepine receptor/Cl- channel complex. J. Neurosci. Off. J. Soc. Neurosci..

[15] Squires R.F., Brastrup C. (1977). Benzodiazepine receptors in rat brain. Nature.

[16] Barkus C., McHugh S.B., Sprengel R., Seeburg P.H., Rawlins J.N., Bannerman D.M. (2010). Hippocampal NMDA receptors and anxiety: At the interface between cognition and emotion. Eur. J. Pharmacol..

[17] Jimenez J.C., Su K., Goldberg A.R., Luna V.M., Biane J.S., Ordek G., Zhou P., Ong S.K., Wright M.A., Zweifel L. (2018). Anxiety Cells in a Hippocampal-Hypothalamic Circuit. Neuron.

[18] Fanselow M.S., Dong H.W. (2010). Are the dorsal and ventral hippocampus functionally distinct structures?. Neuron.

[19] Wilhelmsson U., Bushong E.A., Price D.L., Smarr B.L., Phung V., Terada M., Ellisman M.H., Pekny M. (2006). Redefining the concept of reactive astrocytes as cells that remain within their unique domains upon reaction to injury. Proc. Natl. Acad. Sci. USA.

[20] Malik A.R., Willnow T.E. (2019). Excitatory Amino Acid Transporters in Physiology and Disorders of the Central Nervous System. Int. J. Mol. Sci..

[21] File S.E. (1985). Tolerance to the behavioral actions of benzodiazepines. Neurosci. Biobehav. Rev..

[22] Haigh J.R., Feely M. (1988). Tolerance to the anticonvulsant effect of benzodiazepines. Trends Pharmacol. Sci..

[23] Schmitt U., Luddens H., Hiemke C. (2001). Behavioral analysis indicates benzodiazepine-tolerance mediated by the benzodiazepine binding-site at the GABA(A)-receptor. Prog. Neuro-Psychopharmacol. Biol. Psychiatry.

[24] Gravielle M.C. (2016). Activation-induced regulation of GABAA receptors: Is there a link with the molecular basis of benzodiazepine tolerance?. Pharmacol. Res..

[25] Duke A.N., Tiruveedhula V., Sharmin D., Knutson D.E., Cook J.M., Platt D.M., Rowlett J.K. (2021). Tolerance and dependence following chronic alprazolam treatment in rhesus monkeys: Role of GABA(A) receptor subtypes. Drug Alcohol Depend..

[26] Barker J.S., Hines R.M. (2020). Regulation of GABAA Receptor Subunit Expression in Substance Use Disorders. Int. J. Mol. Sci..

[27] Henry M.E., Jensen J.E., Licata S.C., Ravichandran C., Butman M.L., Shanahan M., Lauriat T.L., Renshaw P.F. (2010). The acute and late CNS glutamine response to benzodiazepine challenge: A pilot pharmacokinetic study using proton magnetic resonance spectroscopy. Psychiatry Res..

[28] Bonavita C., Ferrero A., Cereseto M., Velardez M., Rubio M., Wikinski S. (2003). Adaptive changes in the rat hippocampal glutamatergic neurotransmission are observed during long-term treatment with lorazepam. Psychopharmacology.

[29] Perez M.F., Salmiron R., Ramirez O.A. (2003). NMDA-NR1 and -NR2B subunits mRNA expression in the hippocampus of rats tolerant to Diazepam. Behav. Brain Res..

[30] Tsuda M., Shimizu N., Yajima Y., Suzuki T., Misawa M. (1998). Hypersusceptibility to DMCM-induced seizures during diazepam withdrawal in mice: Evidence for upregulation of NMDA receptors. Naunyn-Schmiedeberg’Pharmacol..

[31] File S.E., Fernandes C. (1994). Dizocilpine prevents the development of tolerance to the sedative effects of diazepam in rats. Pharmacol. Biochem. Behav..

[32] Stephens D.N., Turski L. (1993). Kindling to the benzodiazepine receptor inverse agonist, FG 7142: Evidence for involvement of NMDA, but not non-NMDA, glutamatergic receptors. Neuropharmacology.

[33] Stephens D.N., Andrews J.S., Turski L., Schneider H.H., Briley M., File S.E. (1991). Excitatory Amino Acids and Anxiety. New Concepts in Anxiety.

[34] Palmada M., Kinne-Saffran E., Centelles J.J., Kinne R.K. (2002). Benzodiazepines differently modulate EAAT1/GLAST and EAAT2/GLT1 glutamate transporters expressed in CHO cells. Neurochem. Int..

[35] Sengupta P. (2013). The Laboratory Rat: Relating Its Age With Human’s. Int. J. Prev. Med..

[36] Mitrovic N., Gusevac I., Drakulic D., Stanojlovic M., Zlatkovic J., Sevigny J., Horvat A., Nedeljkovic N., Grkovic I. (2016). Regional and sex-related differences in modulating effects of female sex steroids on ecto-5′-nucleotidase expression in the rat cerebral cortex and hippocampus. Gen. Comp. Endocrinol..

[37] Markwell M.A., Haas S.M., Bieber L.L., Tolbert N.E. (1978). A modification of the Lowry procedure to simplify protein determination in membrane and lipoprotein samples. Anal. Biochem..

[38] Dragic M., Milicevic K., Adzic M., Stevanovic I., Ninkovic M., Grkovic I., Andjus P., Nedeljkovic N. (2021). Trimethyltin Increases Intracellular Ca(2+) Via L-Type Voltage-Gated Calcium Channels and Promotes Inflammatory Phenotype in Rat Astrocytes In Vitro. Mol. Neurobiol..

